# Clinical performance of an innovative self-cure bulk-fill composite compared to dual-cure and light-cure bulk-fill composite in posterior restorations: a 1-year randomized controlled clinical trial

**DOI:** 10.1007/s00784-026-06945-1

**Published:** 2026-06-03

**Authors:** Marwan Abdelnasser Ragab, Waleed A. Elmahy, Marihan Elgayar

**Affiliations:** Conservative Dentistry Department, Faculty of Dentistry, Alexandria University, Alexandria, Egypt

**Keywords:** Light-cure, Self-cure, Dual-cure, Bulk-fill, Clinical performance, Class II

## Abstract

**Objectives:**

To evaluate and compare the 1-year clinical performance of self-cure, dual-cure, and light-cure bulk-fill composites in class II restorations.

**Materials and methods:**

Twenty patients received sixty direct class II restorations, which were divided into three groups (*n* = 20): Group I: Self-cure bulk-fill (Stela capsules) with Stela primer, Group II: Dual-cure bulk-fill (Fill-Up) with ParaBond adhesive system, Group III: Light-cure bulk-fill (Tetric N-Ceram Bulk Fill) with Tetric N-Bond Universal adhesive. Using the Revised FDI criteria, restorations were assessed after 1 week (baseline) and at 3, 6, 9, and 12 months. FDI scores were compared between groups and across different time points using Friedman and Bonferroni post-hoc tests (*P* < 0.05).

**Results:**

All restorations demonstrated acceptable FDI scores, with a 100% survival rate over 12 months. For all functional and biological criteria, no significant differences were observed among the groups. Esthetic differences were observed where Tetric N-Ceram Bulk Fill demonstrated superior surface luster and texture compared with Stela capsules (*P* < 0.05), while Fill-Up showed intermediate performance. Stela capsules and Tetric N-Ceram Bulk Fill exhibited the highest color match scores, whereas Fill-Up was significantly lower (*P* < 0.05). No significant changes were observed over time.

**Conclusions:**

Stela capsules and Fill-Up showed comparable 1-year functional and biological outcomes to Tetric N-Ceram Bulk Fill in class II restorations, while esthetic outcomes were material-dependent.

**Clinical relevance:**

Self- and dual-cure bulk fill composites represent reliable alternatives to light-cure bulk fill materials for posterior restorations where esthetics are of lesser concern, offering simplified placement without compromising short-term clinical performance.

## Introduction

Due to the worldwide agreement to gradually phase out dental amalgam [1, 2], resin-based composites (RBCs) have emerged as a primary alternative for direct restorations [3, 4]. While amalgam offers advantages in ease of handling, the lack of need for costly equipment like light-curing devices, and long-term durability [5], RBCs are favored for their superior esthetics, adhesive capability, and suitability for minimally invasive preparations [6].

Conventional RBCs require incremental placement in layers thinner than 2 mm to allow sufficient light transmission, adequate polymerization, and reduced polymerization shrinkage stresses, which are associated with marginal leakage, postoperative sensitivity, and restoration failure [7]. Although the incremental approach is effective, it is time-consuming and technique-sensitive, which can limit clinical efficiency [8].

Bulk-fill composites (BFCs) were introduced as a significant advancement to address these limitations. They allow application in layers of up to 4 mm, thereby decreasing application time and minimizing the likelihood of void formation or contamination between increments. This simplified bulk-fill application has become a common substitute for the traditional method, especially in deep posterior cavities, with great clinical performance [9, 10].

Material fracture and recurrent decay are the most common causes of failure of RBC restorations [3, 11, 12]. In class II restorations, recurrent decay frequently occurs at the gingival step of the proximal box [12]. This area presents a significant clinical challenge because it is farthest from the source of curing light. As light intensity diminishes rapidly with increased distance from the source, achieving adequate photo-activation quality and monomer conversion rate becomes more difficult in this critical area. Insufficient polymerization compromises both the restorative material and the adhesive interface, resulting in reduced mechanical properties, impaired bonding performance, and ultimately an increased risk of recurrent caries [13–15].

To address the limitations associated with light curing, dual-cure BFCs were introduced. They integrate chemical and light-activated polymerization mechanisms, allowing the restoration surface to be light-cured for finishing and polishing, while the deeper layers polymerize chemically over time [8, 16]. One of the dual-cure BFCs is Fill-Up (Coltene Whaledent AG, Altstatten, Switzerland), which can be light-cured, finished, and polished; meanwhile, the material continues to chemically cure throughout the entire depth within three to four minutes, and as the manufacturer claims, it can be applied in a single layer for cavities up to 10 mm in depth [8, 17]. Beyond this clinical benefit compared with conventional light-cure BFCs, dual-cure BFCs exhibit a slower polymerization process, which may help reduce shrinkage stress [18], offering a promising clinical performance [19].

Another modality of BFCs is a recently introduced self-cure BFC system, Stela (SDI, Bayswater, Victoria, Australia), that is proposed as the true amalgam substitute. Stela BFC is available in auto-mix syringes and capsules, eliminating the need for light activation and providing an unlimited curing depth, making it suitable for restorations exceeding 5 mm in depth. The manufacturer promotes Stela as a two-step system: application of a primer followed by placement of the composite material. When stela contacts its primer, polymerization initiates at the tooth-restoration interface, accelerating the curing process and eliminating the need for light-cured adhesives [20]. One of the attractive features of chemically cured BFCs is their capacity to produce minimal polymerization shrinkage stress [21–24]. Its longer pre-gel stage and slower curing reaction help to minimize stress at the bonded surfaces and limit gap development [23, 25]. Given its simplified application protocol, favorable mechanical properties [26], and promising clinical outcomes [27].

However, comprehensive clinical evidence comparing self-cure BFCs to other established bulk-fill systems (light-cure and dual-cure) is still limited. Therefore, our clinical trial aimed to assess the clinical performance of the three modalities of BFCs: self-cure BFC (Stela capsules), dual-cure BFC (Fill-Up), and light-cure BFC (Tetric N-Ceram Bulk Fill) over 12 months.

The null hypothesis was that self-cure, dual-cure, and light-cure BFCs would have the similar clinical performance with no significant differences as assessed by the Revised Fédération Dentaire Internationale (FDI) evaluation criteria and scoring system [28].

## Materials and methods

### Study design

**Fig. 1:**
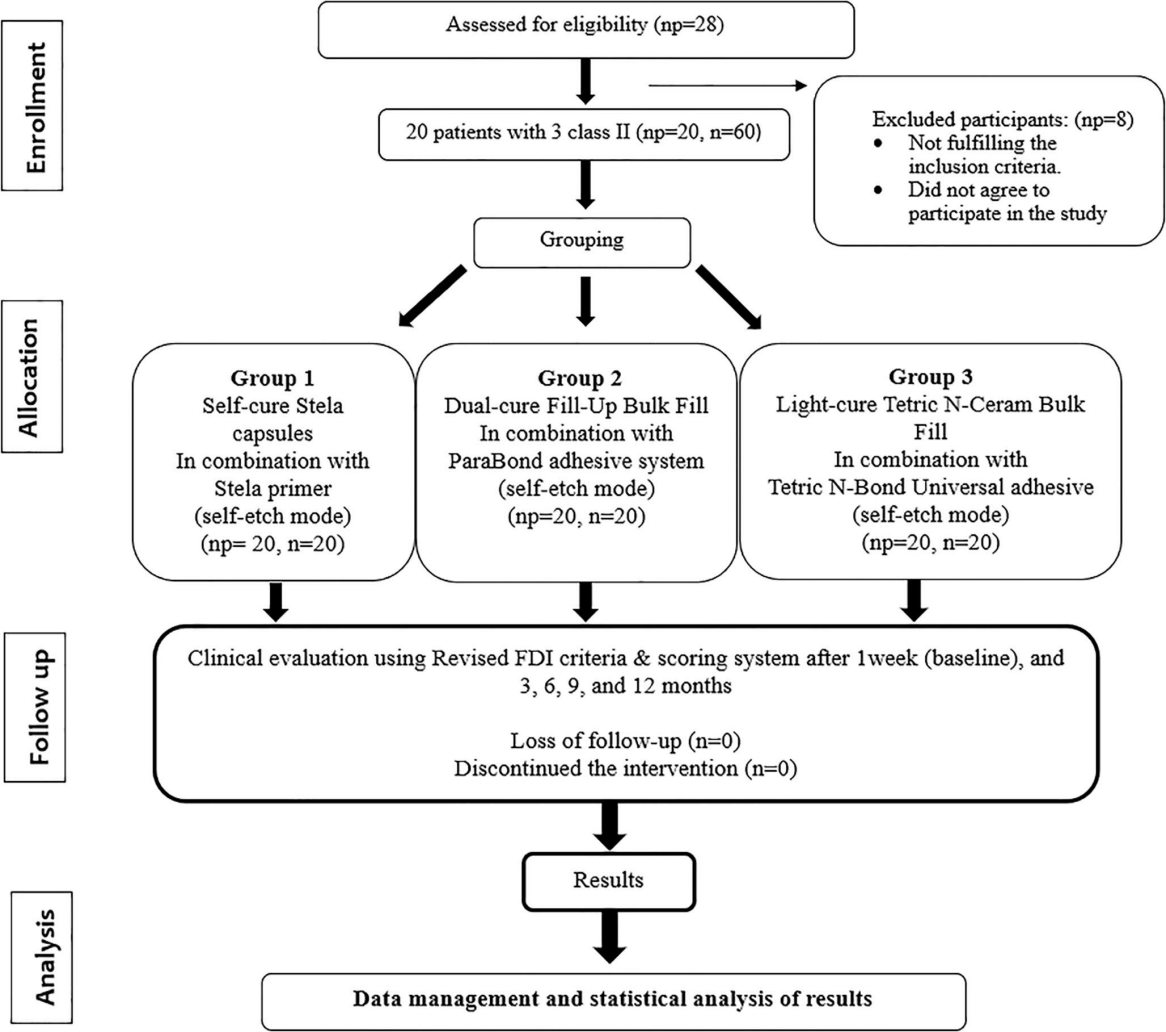
CONSORT flow chart of clinical study. np= number of patients. n= number of teeth

This trial was designed as a randomized controlled clinical trial following the Consolidated Standards of Reporting Trials (CONSORT) guidelines [29] (Fig. 1). Ethical approval was obtained from the Research Scientific Unit Committee of Alexandria University, with reference number (1037-02/2025). The trial was registered at ClinicalTrials.gov as NCT07410468. Before enrollment, detailed information about the study protocol was provided to all participants, who subsequently signed a written consent form. Clinical interventions and follow-up assessments were carried out between February 2025 and February 2026.

### Participants’ recruitment and eligibility criteria

A total of 20 patients were recruited and treated in the Conservative Dentistry Clinics, Faculty of Dentistry, Alexandria University, Alexandria, Egypt. Inclusion criteria required participants to satisfy specific conditions, including: (a) age range between 18 and 45 years; (b) every patient should have at least 3 proximal carious lesions in molars or premolars; (c) all teeth underwent a comprehensive assessment combining clinical examination and radiographic evaluation. Lesion selection was based on clinical assessment, with scores ranging from 2 to 4 according to the International Caries Detection and Assessment System (ICDAS) [30]. In addition, proximal lesions were verified using digital radiographic imaging and were limited to lesion depth not exceeding the middle 1/3 of dentin; (d) Presence of proximal contact with both adjacent and opposing teeth; (e) Absence of previous restorations or clinical signs of pulpitis; (p) Good periodontal health, with no significant systemic diseases or known allergies. Patients who have extensive cavitated lesions, medically compromised, have traumatic malocclusion, bruxism, or parafunctional habits were excluded.

### Sample size calculations

Power analysis was conducted using MedCalc Statistical Software (version 19.0.5; MedCalc Software, Ostend, Belgium) to determine the required sample size. Assuming a 95% confidence interval and an 80% statistical power, the calculated sample size was 18 participants, corresponding to 54 restorations. To account for possible attrition during the follow-up period, the sample was increased to 20 participants, yielding a total of 60 restorations [31]. Accordingly, each of the three study groups included 20 restorations.

### Randomization and allocation concealment

Teeth were randomly allocated into the 3 study groups. Using a permuted block randomization technique, the allocation sequence was generated using computer- generated random allocation software (Sealed Envelope Ltd., London, UK).

Allocation concealment was ensured using opaque, sealed envelopes prepared by a staff member who had no involvement in the study. Each envelope was opened at the time of treatment to assign the restorative material.

Furthermore, both Patients and follow-up examiners were blinded. Therefore, the study has been conducted as a double-blind randomized clinical trial.

### Intervention

#### Cavity preparation

Local anesthesia (Artinibsa 4% 1:100.000, Inibsa Dental S.L.U, Spain) was administered. A wooden wedge was placed interproximally at the surface to be restored. Wedges were placed tightly to prevent damage to the papilla during the entire preparation procedure. Preparation of a class II cavity was established using a high-speed handpiece (TE-95RM W&H Austria) with a sterile diamond bur (FG 108-009 (Komet 835) Horico, Hamburg, Germany), ensuring sufficient water cooling. During preparation, Wedge Guard (Palodent Plus) was used to protect the adjacent tooth during opening the proximal contact. A low-speed handpiece, round steel burs, and hand excavators (Dentsply, Maillefer, Switzerland) were used to remove any remaining caries.

#### Isolation and matricing

**Fig. 2:**
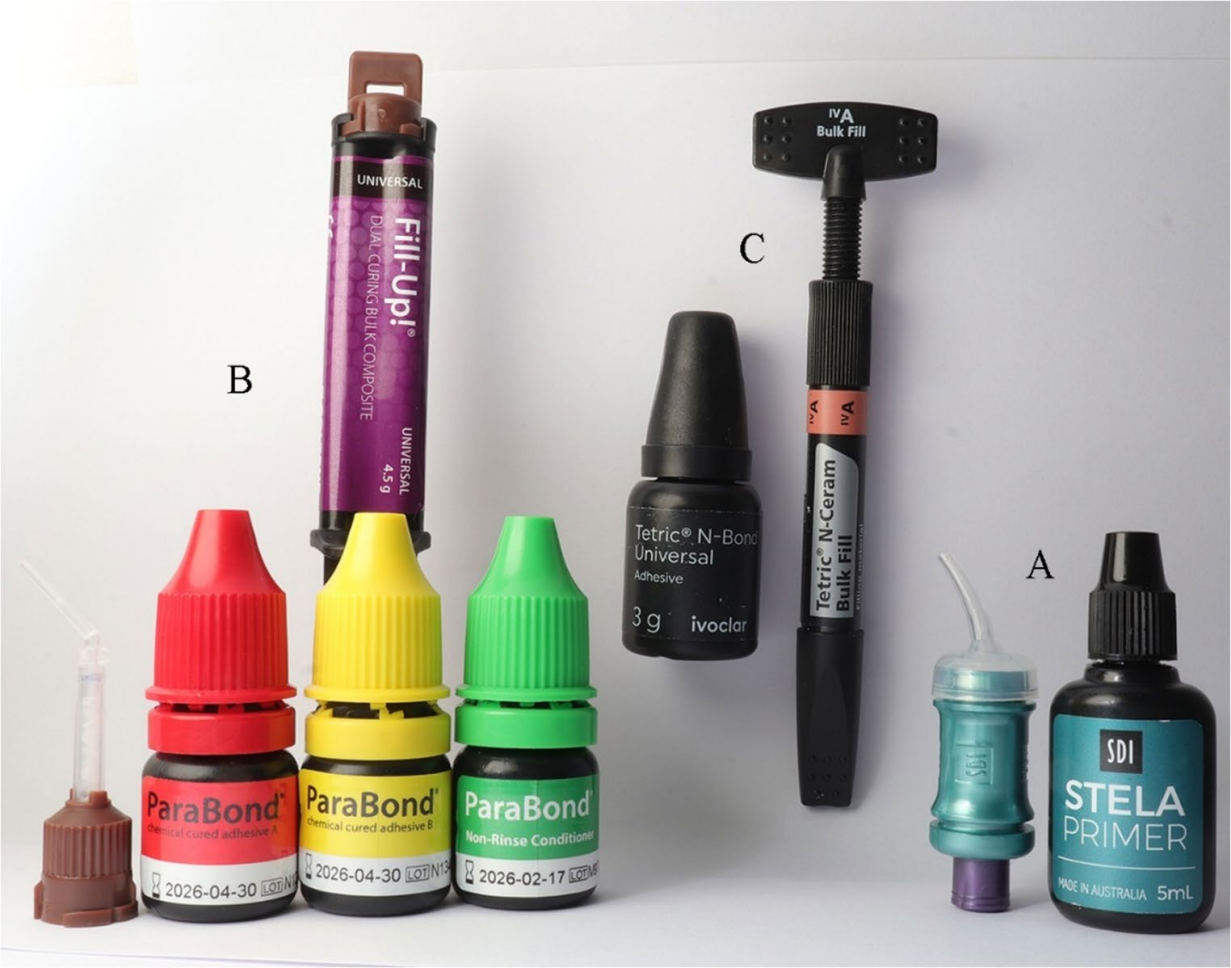
The restorative system used for each study group. **A**: Stela capsules and Stela primer. **B**: Fill-Up dual syringe with ParaBond chemical-cure adhesive system. **C**: Tetric N-Ceram Bulk Fill with Tetric N-Bond Universal Adhesive

After cavity preparation, full isolation using a rubber dam (Nic Tone, MDC Dental, Jalisco, Mexico) was used to avoid any moisture contamination during restorative procedures. The Palodent V3 sectional matrix system and its separating ring and interdental wedges (Palodent V3 Sectional Matrix System, Dentsply Sirona, USA) were used for restoration placement. No liner was used in the prepared cavities. After preparation of the cavity and rubber dam isolation, the sealed envelopes were opened to reveal the restorative material allocated for each tooth. Each restoration was applied following the manufacturer’s instructions, and each cavity was filled with one of the tested BFC materials (Fig. 2).

#### Restoration placement

**Fig. 3:**
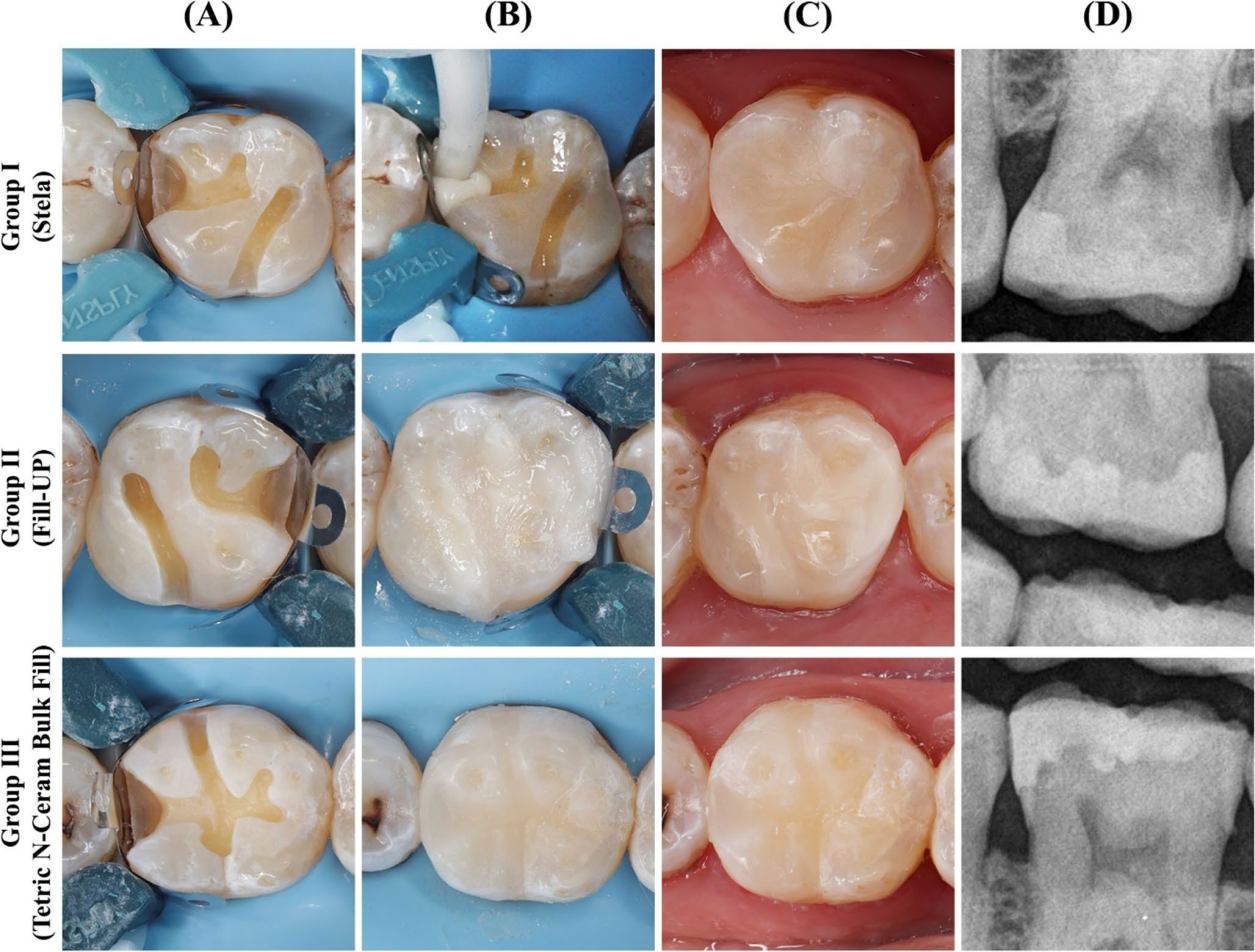
The clinical procedures for the three study groups. (**A**): Prepared cavity with placement of the sectional matrix system and the separating ring. (**B**): Restorative material placement according to each group. (**C**): Finished and polished restoration. (**D**): Postoperative radiograph

For Group 1, Stela chemical cure capsules were used with Stela primer (SDI, Victoria, Australia) (Fig. 2A). Two drops of Stela Primer were placed into a mixing well. The primer was thoroughly applied to cavity surfaces and margins for 10 s using an applicator brush (Points, SDI) with vigorous agitation. It was subsequently left undisturbed for 5 s, after which gentle air-drying was performed for 3 s to promote solvent evaporation and achieve an even primer layer. The Stela capsule was activated by depressing the plunger and subsequently mixed for 10 s using an Ultramat 2 triturator (SDI, Victoria, Australia). Following mixing, the capsule was inserted into the SDI applicator, which was activated by clicking the trigger until the material became visible through the transparent nozzle of the capsule. The nozzle was placed at the gingival step of the primed class II cavity. The material was injected while the capsule tip was slowly moved in an occlusal direction to ensure precise and uniform adaptation to the cavity walls. The restorative material was dispensed into the cavity in one layer, with slight overfilling of cavity margins. The material was carefully contoured without removing any excess from the margins before complete polymerization. Polymerization was allowed to proceed for 4 min after mixing without any light-curing process, after which the restoration was finished and polished (Fig. 3 Group I).

For Group 2, Fill-Up dual-cure BFC was used with ParaBond adhesive system (Fig. 2B). The non-rinse conditioner was applied to the cavity and gently agitated for 30 s using a disposable brush, followed by air-drying for 5 s. Subsequently, one drop of Adhesive A and Adhesive B was mixed in a plastic well, applied to all prepared surfaces, and agitated for 30 s before gentle air-drying. Fill-Up was then injected into the cavity using a dual-chamber syringe with an auto-mix tip, filling the preparation in a single bulk increment from the deepest point, and light-cured for 20 s using LED curing light (Radii Plus, SDI Ltd, Australia) with an output of 1500 W/cm2 (Fig. 3 Group II).

For Group 3, Tetric N-Ceram BFC was used with Tetric N-Bond Universal (Fig. 2C). Bond was gently agitated for 30 s, air-dried, then light-cured for 20 s. Tetric N-Ceram Bulk Fill was applied in up to 4 mm layers. Each layer was light-cured for 20 s. After removal of the matrix, the proximal box of the restorations was also light-cured from the buccal and lingual/palatal aspects for 10 s each (Fig. 3 Group III).

#### Finishing and polishing

Anatomical contouring was performed using fine and extra-fine diamond burs under water cooling to efficiently shape the occlusal anatomy without inducing thermal damage. For embrasure refinement, a No. 12 scalpel blade was used to access interproximal zones and remove excess composite material with precision. Polishing was performed using kerr polishing system (Kerr Dental, Orange, CA, USA), starting with OptiDiscs in a sequential manner from coarse to ultra-fine grit under light pressure and continuous water irrigation. To enhance surface gloss, ProGloss one-step polishing points (Kerr Dental, Orange, CA, USA) were applied to flat and cervical surfaces without additional polishing paste. Final polishing was completed using OptiShine brush (Kerr Dental, Orange, CA, USA), particularly in occlusal grooves and embrasure areas, to refine surface texture and improve luster. Finishing and polishing protocols were performed the same way for all groups.

As Tetric N-Ceram Bulk Fill is a light-cured composite, sufficient working time was available to contour and refine the restoration before polymerization. Therefore, the anatomical form of the restoration was established while the material remained uncured. In contrast, the self- and dual-cured materials provided limited working time before the onset of auto-polymerization, requiring the cavities to be slightly overfilled prior to achieving the final anatomical contour.

#### Calibration, blinding, and follow-up examination

All restorative treatments were carried out by a single clinician. Clinical evaluations were conducted by two previously calibrated examiners who were blinded to the tooth allocation and had no involvement in the restorative treatments. Intra- and inter-examiner reliability was determined using the Intraclass Correlation Coefficient (ICC), demonstrating good agreement, with values ranging from 0.71 to 0.83. The outcome assessors remained blinded to the prior interventions, and the clinician only identified the tooth under evaluation without disclosing the material used. Clinical evaluations were performed using mouth mirrors and probes, supplemented by bite-wing x-rays and intraoral photographs. In instances of scoring discrepancies between the two examiners, the final score was determined through consensus.

All restorations were assessed clinically at 1 week (baseline) and subsequently at 3, 6, 9, and 12 months, following the revised FDI evaluation criteria and scoring system [28], which indicates the functional, biological, and aesthetic properties of the examined restorative materials.

Each evaluated property was scored according to the FDI criteria, where score 1: Clinically excellent/very good (VG), score 2: clinically good (CG), score 3: clinically satisfactory (CS), score 4: clinically unsatisfactory (CU), and score 5: clinically poor (PO).

### Statistical analysis

Descriptive data were summarized as percentages and frequencies. Comparisons of FDI criteria between the three groups and across different timepoints within each group were performed using Friedman test. When significant differences were detected, multiple pairwise comparisons were conducted with Bonferroni correction. An intention-to-treat analysis was applied throughout the analysis. Significance level was set at p-value < 0.05. Data were analyzed using IBM SPSS for Windows (Version 26.0).

## Results

All patients were available for evaluation at all follow-up periods, and no dropouts were recorded during the 12-month observation period, resulting in a 100% recall rate at all follow-up intervals. Sample description and distribution are shown in Tables 1 and 2.

**Table 1:** Sample description (np = 20)

		np (%)
Gender	**Male**	7 (35%)
	**female**	13 (65%)
Age	**18–29**	12 (60%)
	**30–39**	6 (30%)
	**40–45**	2 (10%)

**Table 2:** Characteristics and distribution of restorations within each group

		Group I (*n* = 20)	Group II (*n* = 20)	Group III (*n* = 20)	*P* value
Presence of antagonists	**Yes**	20 (100%)	20 (100%)	20 (100%)	1.00
	**No**	-	-	-	
Teeth distribution	**Upper premolar**	5 (25%)	4 (20%)	7 (35%)	0.61
	**Lower premolar**	7 (35%)	4 (20%)	3 (15%)	
	**Upper molar**	5 (25%)	5 (25%)	4 (20%)	
	**Lower molar**	3 (15%)	7 (35%)	6 (30%)	
Number of restored surfaces	**1**	-	-	-	1.00
	**2**	17 (85%)	17 (85%)	17 (85%)	
	**3**	3 (15%)	3 (15%)	3 (15%)	
	**≥ 4**	-	-	-	
Reasons for restoration	**Primary caries**	20 (100%)	20 (100%)	20 (100%)	1.00
	**Secondary caries**	-	-	-	
	**Restoration replacement**	-	-	-	

### Functional criteria (FDI category F)

After a 12-month follow-up period, all restorations in the three groups remained intact and fully retained. No fracture or complete/partial losses of restorations (F1), loss of proximal contact point (F3), deviation from ideal anatomic form and contour (F4), clinically detectable occlusal discrepancies, or abnormal wear patterns (F5) were encountered with 100% of restorations rated as Score 1 at all evaluation intervals. Regarding marginal adaptation (F2) starting from 6 months, minor marginal discrepancies (Score 2) were observed. These differences were not statistically significant at any time point (*p* > 0.05). (Table 3).

### Biological criteria (FDI category B)

Throughout the study period, no restorations were detected with recurrence of caries (B1) or dental hard tissue defects or fractures (B2) at the restoration margins in the three groups. Only at baseline, slight postoperative sensitivity (Score 2) was reported with no statistically significant differences between groups. (Table 4).

### Aesthetic criteria (FDI category A)

**Fig. 4:**
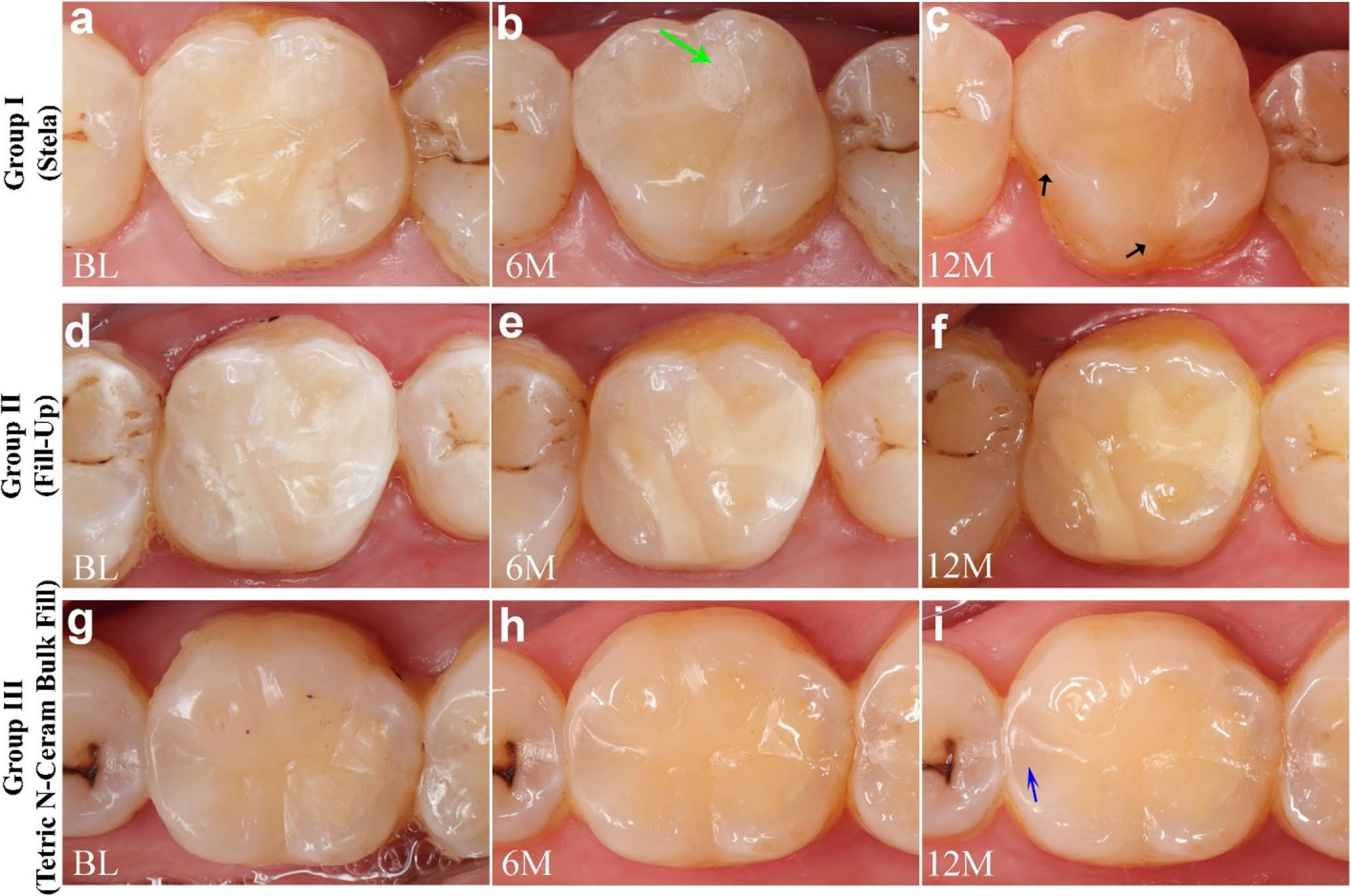
The clinical follow-ups across 12 months. The upper row (**a**-**c**): group I, the middle row (**d**-**f**): group II, and the lower row (**g**-**i**): group III. Note the superior surface luster and texture of group III (Tetric N-Ceram Bulk Fill). Note the inferior color matching and increased opacity of group II (Fill-Up). Black arrows: minor marginal staining. The green arrow: some isolated pores. The blue arrow: increased translucency and a greyish shadow of the composite at the proximal box area

Regarding surface luster and texture (A1), post-hoc analysis revealed significant differences mainly between Group I and Group III at all evaluation intervals (*p* < 0.05), favoring the light-cured bulk fill over the self-cured bulk fill. Group I exhibited the highest proportion of restorations rated as clinically good but not ideal restorations (Score 2: 40%), and was the only group presenting clinically satisfactory/acceptable restorations (Score 3: 10%), which appeared as minor isolated pores (Fig. 4; green arrow). This pattern remained consistent throughout the follow-up periods at 3, 6, 9, and 12 months (Table 5).

In terms of marginal staining (A2), starting from 9 months, minor marginal staining (Score 2) was observed in Group I and II restorations. These differences were not statistically significant at any time point (*p* > 0.05).

Concerning color match (A3), Group I showed better color match than Group II at all time points (*p* < 0.05). At 12 months, Group III also showed a better color match than Group II (*p* < 0.05). Group I showed better scores than Group III, but no statistically significant differences were detected between them at any evaluation period. Deviation in color match in group III restorations was presented mainly as increased translucency of the material, especially at the marginal ridge of class II restoration when compared to that of the adjacent natural tooth (Fig. 4; blue arrow). In contrast, Group II (Fill-Up) exhibited the lowest percentage of restorations with excellent color match (Score 1), and was the only group in which Score 3 was observed. This deviation in color match was observed as a minor to distinct increase in opacity of the restoration, accompanied by diminished translucency (Fig. 4; d-f). Within each group, color match remained stable over time, with no significant intragroup changes during the 12-month follow-up (*p* > 0.05).

**Table 3:** Comparison of FDI functional criteria between the three study groups

		Group I					Group II					Group III					*P* value 1
		1	2	3	4	5	1	2	3	4	5	1	2	3	4	5	
		N (%)															
F1	**baseline**	20 (100%)	-	-	-	-	20 (100%)	-	-	-	-	20 (100%)	-	-	-	-	1.00
	**3M**	20 (100%)	-	-	-	-	20 (100%)	-	-	-	-	20 (100%)	-	-	-	-	1.00
	**6M**	20 (100%)	-	-	-	-	20 (100%)	-	-	-	-	20 (100%)	-	-	-	-	1.00
	**9M**	20 (100%)	-	-	-	-	20 (100%)	-	-	-	-	20 (100%)	-	-	-	-	1.00
	**12M**	20 (100%)	-	-	-	-	20 (100%)	-	-	-	-	20 (100%)	-	-	-	-	1.00
	**P value 2**	1.00					1.00					1.00					
F2	**Baseline**	20 (100%)	-	-	-	-	20 (100%)	-	-	-	-	20 (100%)	-	-	-	-	1.00
	**3M**	20 (100%)	-	-	-	-	20 (100%)	-	-	-	-	20 (100%)	-	-	-	-	1.00
	**6M**	19 (95%)	1 (5%)	-	-	-	18 (90%)	2 (10%)	-	-	-	20 (100%)	-	-	-	-	0.36
	**9M**	19 (95%)	1 (5%)	-	-	-	18 (90%)	2 (10%)	-	-	-	20 (100%)	-	-	-	-	0.36
	**12M**	19 (95%)	1 (5%)	-	-	-	18 (90%)	2 (10%)	-	-	-	19 (95%)	1 (5%)	-	-	-	0.78
	**P value 2**	0.41					0.10					0.10					
F3	**Baseline**	20 (100%)	-	-	-	-	20 (100%)	-	-	-	-	20 (100%)	-	-	-	-	1.00
	**3M**	20 (100%)	-	-	-	-	20 (100%)	-	-	-	-	20 (100%)	-	-	-	-	1.00
	**6M**	20 (100%)	-	-	-	-	20 (100%)	-	-	-	-	20 (100%)	-	-	-	-	1.00
	**9M**	20 (100%)	-	-	-	-	20 (100%)	-	-	-	-	20 (100%)	-	-	-	-	1.00
	**12M**	20 (100%)	-	-	-	-	20 (100%)	-	-	-	-	20 (100%)	-	-	-	-	1.00
	**P value 2**	1.00					1.00					1.00					
F4	**Baseline**	20 (100%)	-	-	-	-	20 (100%)	-	-	-	-	20 (100%)	-	-	-	-	1.00
	**3M**	20 (100%)	-	-	-	-	20 (100%)	-	-	-	-	20 (100%)	-	-	-	-	1.00
	**6M**	20 (100%)	-	-	-	-	20 (100%)	-	-	-	-	20 (100%)	-	-	-	-	1.00
	**9M**	20 (100%)	-	-	-	-	20 (100%)	-	-	-	-	20 (100%)	-	-	-	-	1.00
	**12M**	20 (100%)	-	-	-	-	20 (100%)	-	-	-	-	20 (100%)	-	-	-	-	1.00
	**P value 2**	1.00					1.00					1.00					
F5	**Baseline**	20 (100%)	-	-	-	-	20 (100%)	-	-	-	-	20 (100%)	-	-	-	-	1.00
	**3M**	20 (100%)	-	-	-	-	20 (100%)	-	-	-	-	20 (100%)	-	-	-	-	1.00
	**6M**	20 (100%)	-	-	-	-	20 (100%)	-	-	-	-	20 (100%)	-	-	-	-	1.00
	**9M**	20 (100%)	-	-	-	-	20 (100%)	-	-	-	-	20 (100%)	-	-	-	-	1.00
	**12M**	20 (100%)	-	-	-	-	20 (100%)	-	-	-	-	20 (100%)	-	-	-	-	1.00
	**P value 2**	1.00					1.00					1.00					

*P* value 1: Comparisons between groups using Friedman test, P value 2: Comparisons between timepoints within each group using Friedman test

**Table 4:** Comparison of FDI biological criteria between the three study groups

		Group I					Group II					Group III					*P* value 1
		1	2	3	4	5	1	2	3	4	5	1	2	3	4	5	
		N (%)															
B1	**Baseline**	20 (100%)	-	-	-	-	20 (100%)	-	-	-	-	20 (100%)	-	-	-	-	1.00
	**3M**	20 (100%)	-	-	-	-	20 (100%)	-	-	-	-	20 (100%)	-	-	-	-	1.00
	**6M**	20 (100%)	-	-	-	-	20 (100%)	-	-	-	-	20 (100%)	-	-	-	-	1.00
	**9M**	20 (100%)	-	-	-	-	20 (100%)	-	-	-	-	20 (100%)	-	-	-	-	1.00
	**12M**	20 (100%)	-	-	-	-	20 (100%)	-	-	-	-	20 (100%)	-	-	-	-	1.00
	**P value 2**	1.00					1.00					1.00					
B2	**Baseline**	20 (100%)	-	-	-	-	20 (100%)	-	-	-	-	20 (100%)	-	-	-	-	1.00
	**3M**	20 (100%)	-	-	-	-	20 (100%)	-	-	-	-	20 (100%)	-	-	-	-	1.00
	**6M**	20 (100%)	-	-	-	-	20 (100%)	-	-	-	-	20 (100%)	-	-	-	-	1.00
	**9M**	20 (100%)	-	-	-	-	20 (100%)	-	-	-	-	20 (100%)	-	-	-	-	1.00
	**12M**	20 (100%)	-	-	-	-	20 (100%)	-	-	-	-	20 (100%)	-	-	-	-	1.00
	**P value 2**	1.00					1.00					1.00					
B3	**Baseline**	20 (100%)	-	-	-	-	16 (80%)	3 (15%)	1 (5%)	-	-	19 (95%)	1 (5%)	-	-	-	0.08
	**3M**	20 (100%)	-	-	-	-	20 (100%)	-	-	-	-	20 (100%)	-	-	-	-	1.00
	**6M**	20 (100%)	-	-	-	-	20 (100%)	-	-	-	-	20 (100%)	-	-	-	-	1.00
	**9M**	20 (100%)	-	-	-	-	20 (100%)	-	-	-	-	20 (100%)	-	-	-	-	1.00
	**12M**	20 (100%)	-	-	-	-	20 (100%)	-	-	-	-	20 (100%)	-	-	-	-	1.00
	**P value 2**	1.00					0.32					0.41					

*P* value 1: Comparisons between groups using Friedman test, P value 2: Comparisons between timepoints within each group using Friedman test

**Table 5:** Comparison of FDI aesthetic criteria between the three study groups

		Group I					Group II					Group III					*P* value 1
		1	2	3	4	5	1	2	3	4	5	1	2	3	4	5	
		*N* (%)															
A1	**Baseline**	10 (50%) **a**	8 (40%)	2 (10%)	-	-	14 (70%) **ab**	6 (30%)	-	-	-	19 (95%) **b**	1 (5%)	-	-	-	0.003*
	**3M**	10 (50%) **a**	8 (40%)	2 (10%)	-	-	14 (70%) **ab**	6 (30%)	-	-	-	19 (95%) **b**	1 (5%)	-	-	-	0.003*
	**6M**	11 (55%) **a**	8 (40%)	1 (5%)	-	-	14 (70%) **ab**	6 (30%)	-	-	-	19 (95%) **b**	1 (5%)	-	-	-	0.006*
	**9M**	10 (50%) **a**	8 (40%)	2 (10%)	-	-	14 (70%) **ab**	6 (30%)	-	-	-	19 (95%) **b**	1 (5%)	-	-	-	0.003*
	**12M**	11 (55%) **a**	7 (35%)	2 (10%)	-	-	14 (70%) **ab**	6 (30%)	-	-	-	19 (95%) **b**	1 (5%)	-	-	-	0.005*
	**P value 2**	0.17					1.00					1.00					
A2	**Baseline**	20 (100%)	-	-	-	-	20 (100%)	-	-	-	-	20 (100%)	-	-	-	-	1.00
	**3M**	20 (100%)	-	-	-	-	20 (100%)	-	-	-	-	20 (100%)	-	-	-	-	1.00
	**6M**	20 (100%)	-	-	-	-	20 (100%)	-	-	-	-	20 (100%)	-	-	-	-	1.00
	**9M**	19 (95%)	1 (5%)	-	-	-	18 (90%)	2 (10%)	-	-	-	20 (100%)	-	-	-	-	0.36
	**12M**	18 (90%)	2 (10%)	-	-	-	18 (90%)	2 (10%)	-	-	-	20 (100%)	-	-	-	-	0.35
	**P value 2**	0.17					0.10					1.00					
A3	**Baseline**	18 (90%) **a**	2 (10%)	-	-	-	10 (50%) **b**	8 (40%)	2 (10%)	-	-	15 (75%) **ab**	5 (25%)	-	-	-	0.02*
	**3M**	18 (90%) **a**	2 (10%)	-	-	-	10 (50%) **b**	8 (40%)	2 (10%)	-	-	14 (70%) **ab**	6 (30%)	-	-	-	0.03*
	**6M**	18 (90%) **a**	2 (10%)	-	-	-	10 (50%) **b**	8 (40%)	2 (10%)	-	-	14 (70%) **ab**	6 (30%)	-	-	-	0.03*
	**9M**	18 (90%) **a**	2 (10%)	-	-	-	10 (50%) **b**	8 (40%)	2 (10%)	-	-	15 (75%) **ab**	5 (25%)	-	-	-	0.02*
	**12M**	17 (85%) **a**	3 (15%)	-	-	-	8 (40%) **b**	9 (45%)	3 (15%)	-	-	16 (80%) **a**	4 (20%)	-	-	-	0.008*
	**P value 2**	0.41					0.45					0.23					

*P* value 1: Comparisons between groups using Friedman test

*P* value 2: Comparisons between timepoints within each group using Friedman test

*statistically significant at p-value < 0.05

a-b: different lowercase letters denote significant differences between groups using Bonferroni correction

## Discussion

In light of the previous findings, the null hypothesis was accepted as all three materials demonstrated clinically successful short-term performance, with no restoration failures and predominantly ideal scores in the functional and biological domains, and only minor differences in the aesthetic parameters. These findings support the clinical feasibility of BFC placement in posterior restorations and confirm that self- and dual-cure bulk-fill can provide outcomes comparable to light-cure BFCs.

Based on the available literature, this study represents the first clinical trial to compare the clinical performance of one of each class of BFCs: self, dual, and light-cure BFCs in posterior class II restorations. In this study, Restorations were assessed at each follow-up recall using the revised FDI criteria and scoring system [28]. This method of assessment was chosen over the United States Public Health Service (USPHS) criteria [32]. Due to its noted higher sensitivity in detecting early pathological changes [33–36].

From a functional standpoint, restorations were excellent across all groups. These findings align with earlier randomized clinical trials, which have demonstrated high survival rates and favorable mechanical performance of BFCs in posterior restorations [10, 19, 27]. The absence of catastrophic failures suggests that the mechanical characteristics of the examined restorations are sufficient to withstand occlusal loading in class II cavities [37–39]. Supporting this, an in vitro investigation [40] showed that Stela capsules and Fill-up exhibited acceptable flexural strength, surface hardness, and solubility, and that their mechanical properties were comparable to those of light-cured RBCs [41, 42].

Marginal adaptation remained stable, with only minor, non-significant discrepancies (score 2) observed in a small number of restorations at later recalls. This finding aligns with the small degree of marginal discoloration detected in some restorations, which may indicate minor adhesive degradation and the development of minute marginal gaps [43, 44]. Nevertheless, all restorations were rated as FDI score 1 or 2 at 12 months, confirming their continued clinical acceptability and suggesting that the minor reduction in marginal integrity did not compromise overall performance or durability.

Regarding biological performance, all three bulk-fill systems demonstrated excellent behavior throughout the 12-month follow-up period. Postoperative sensitivity was minimal and transient. Previous clinical studies have shown that hypersensitivity may occur in the few days or weeks following restorative procedures; the symptoms typically subside rapidly during this period. Therefore, these episodes of hypersensitivity are more likely to be caused by restorative procedures such as caries excavation, cavity depth, rubber dam placement, cavity drying, bonding strategy, technique-dependent factors, photo-activation efficiency, or pre-existing pulpal status, than by restorative materials [45–47]. The absence of persistent sensitivity in the present study suggests that all three materials provided adequate stress management during polymerization and effective sealing of dentin.

BFCs are designed to reduce polymerization shrinkage stress through different mechanisms, such as modified resin matrices, stress-relieving monomers, and optimized filler loading [8, 48]. Excessive shrinkage stress has been strongly associated with gap formation, microleakage, postoperative sensitivity, and ultimately recurrent decay [7, 22, 49]. In previous studies [50, 51], Self and dual-cured materials exert lower stress than their light-cured counterparts due to the slower polymerization kinetics, which typically generates lower stress due to the extended pre-gel phase, allowing for stress relaxation before network vitrification. Laboratory investigations have also demonstrated improved depth of cure with more homogenous polymerization for chemical and dual-cure bulk fill materials [40, 52, 53], which is particularly advantageous in deep class II cavities where light penetration is limited [50].

An in-vitro study [20] showed that Stela BFC and its associated primer exhibit a distinctive pattern of polymerization initiation based on touch-cure polymerization mode, where polymerization was initiated by the Stela Primer on the cavity walls and floor. Consequently, curing began at the cavity walls rather than within the composite core. This interfacial mode of initiation may enhance adaptation to cavity walls and reduce interfacial gap formation [24, 54]. In contrast, Tetric N-Ceram Bulk Fill mitigates shrinkage stress through intrinsic material design. Jang et al. [55] demonstrated that, as the manufacturer states, Tetric N-Ceram Bulk Fill has a “shrinkage stress reliever,” a specially engineered filler with a low elastic modulus that functions as a microscopic spring, dissipating contraction forces during curing. Although these materials rely on different mechanisms (polymerization kinetics versus structural stress relief), both strategies appear clinically effective, as reflected by the absence of persistent postoperative sensitivity observed in our study.

The adhesive interface also has a critical role in the biological performance of the restoration [56, 57]. In the present study, each bulk-fill system was applied using its manufacturer-recommended adhesive protocol, which reflects real clinical practice. Although different adhesive systems were used in the three groups, all of them belong to the self-etch category, which preserves the smear layer and reduces dentinal fluid movement, thereby minimizing postoperative sensitivity and enhancing marginal sealing [58]. Furthermore, the relation between polymerization shrinkage stress and adhesive performance is critical. Even low-shrinkage bulk-fill composites can generate stresses that challenge the adhesive interface, particularly in high C-factor class II cavities. An adhesive system with insufficient bond strength or elastic buffering capacity may fail to maintain marginal integrity under these stresses [59]. The absence of marginal breakdown and dental defects in our study implies that the bonding agents used in all groups were capable of withstanding these stresses, contributing substantially to the excellent biological performance observed. These findings highlight that biological success depends on the combined performance of the restorative material and adhesive system rather than the composite alone.

A particular concern with deep posterior restorations is insufficient polymerization at the cavity floor and gingival step, especially when light-cured materials are used in areas where light transmission is limited [60]. Inadequate curing may compromise mechanical properties, increase solubility, and adversely affect biocompatibility, potentially leading to pulpal irritation and bacterial penetration [61]. The present findings suggest that both self-cure and dual-cure systems were able to overcome this limitation, achieving biological outcomes comparable to those of light-cure BFCs.

The absence of secondary caries across all groups also aligns with previous clinical trials on bulk-fill composites, which have reported low caries incidence during short- and medium-term follow-up [19, 27, 62].

Collectively, these findings indicate that the curing mechanism—whether self-, dual-, or light-activated—did not negatively affect the biological performance of class II restorations over one year. The results support the clinical safety of newer self-cure and dual-cure bulk-fill systems and suggest that they can provide an effective marginal seal and pulpal protection comparable to established light-cured materials. However, as biological failures such as secondary caries and pulpal pathology often develop over longer periods, extended follow-up is required to confirm whether these favorable outcomes are maintained in the long term.

In contrast to the uniform biological and functional outcomes, aesthetic variations between materials were encountered. Tetric N-Ceram Bulk Fill showed superior surface gloss and texture compared with Stela, while Fill-Up showed intermediate performance. Similar outcomes have been documented in previous clinical trials [19, 27]. Surface gloss and its long-term maintenance are strongly influenced by filler characteristics, especially particle size, morphology, and loading, which affect polishability and resistance to surface degradation. Composites with smaller and more uniformly distributed fillers achieve smoother surfaces and better gloss retention. Whereas Larger and irregular filler particles are more susceptible to dislodgement during finishing and polishing, resulting in surface pitting and irregularities [63].

These findings may be attributed to the different filler technologies of the investigated materials. Tetric N-Ceram Bulk Fill is a nano-hybrid BFC, whereas Stela and Fill-Up are micro-hybrid. Nano-hybrid composites incorporate finer filler particles that allow for more homogeneous surface abrasion and enhanced gloss stability [64]. Although both Stela and Fill-Up are classified as micro-hybrids, they differ in filler characteristics: Stela contains fillers with a mean size of approximately 4 μm and a filler loading of 76.8 wt%, whereas Fill-Up contains smaller fillers, approximately 2 μm, with a lower filler loading (65 wt%) Previous study indicate that smaller filler size and higher filler loading reduce surface roughness [65], explaining the intermediate polishability of Fill-Up and the lower surface luster of Stela.

Regarding color match, Stela demonstrated the best and most consistent shade integration, followed by Tetric N-Ceram Bulk Fill, while Fill-Up showed inferior performance. The superior shade matching of Stela can be attributed to its fluorine-alumino-silicate glass fillers with a controlled particle size distribution (approximately 2–8 μm, median ~ 4 μm) and a high filler loading (≈ 76.8 wt%). This relatively homogenous filler system enhances refractive index matching between the resin and fillers and reduces internal light scattering. In contrast, Fill-Up incorporates zinc-oxide-coated fillers with a broader particle size distribution (0.1–5 μm) and a lower filler loading (≈ 65 wt%). The high refractive index of zinc oxide increases opacity and light scattering, negatively affecting translucency and color blending. These findings support previous evidence that filler type, size, and refractive index compatibility are key determinants of color integration in resin composites [64, 66].

Tetric N-Ceram Bulk-Fill showed good color match, but its increased translucency – designed to improve light transmission to deeper layers [67] - may reduce masking ability where an increased bulk of material is used, such as in proximal box areas, resulting in restorations exhibiting a slight grayish hue at the marginal ridge of class II restorations.

Importantly, none of the three groups exhibited significant color deterioration over time. indicates good short-term color stability and supports previous findings that modern bulk-fill composites exhibit acceptable color stability in the oral environment [19, 27]. Clinically, while all materials were functionally and biologically reliable, Stela and Tetric N-Ceram bulk fill may provide superior color integration in cases with higher esthetic demands, particularly in visible premolar regions.

Several limitations of the present study must be considered. Although the 12-month follow-up was sufficient to detect early failures and postoperative complications, it does not allow assessment of long-term wear, marginal degradation, or late color changes; therefore, longer observation periods are required. The sample size was adequate for short-term evaluation, but it may not capture infrequent biological or mechanical failures. The inability to apply operator blinding because of the use of distinct restorative procedures may have introduced bias into the study. The exclusion of patients with systemic conditions, traumatic occlusion, or pre-existing restorations may restrict the generalizability of the findings to broader patient populations, and the inclusion of carious lesions limited to ICDAS scores 2 to 4 may have influenced cavity selection; further studies should evaluate deeper lesions and their effect on pulp vitality. In addition, all adhesive systems were used in the self-etch mode to ensure standardization, particularly because Stela Primer is recommended exclusively for this mode of application. Although selective enamel etching has been suggested to improve adhesive longevity, recent evidence in posterior restorations supports the findings of our study [68]; nevertheless, further clinical trials are needed to assess the effect of selective enamel etching on the bonding capability of Stela Primer. The simplified placement protocols associated with these materials may reduce chair time and technique sensitivity, but long-term randomized clinical studies are required to verify if these short-term findings can be translated into comparable long-term survival and esthetic stability.

## Conclusions

Within the limitations of the present study, Stela capsules and Fill-Up demonstrated comparable 1-year functional and biological performance to Tetric N-Ceram Bulk Fill in class II restorations. Minor esthetic differences were observed; Tetric N-Ceram Bulk Fill exhibited the highest surface luster, while Fill-Up exhibited the lowest color match among the tested groups.

## Data Availability

No datasets were generated or analysed during the current study.
